# Autoimmune Hemolytic Anemia Following Intravenous Immunoglobulin in Kawasaki Disease

**DOI:** 10.7759/cureus.109047

**Published:** 2026-05-17

**Authors:** Itzamar Pastrana Echevarria, Jesus García, Gabriela M Henriquez, Yesabeli Condor, Vylma Velazquez

**Affiliations:** 1 Medicine, Hospital Episcopal San Lucas, Ponce, PRI; 2 Pediatrics, Hospital Episcopal San Lucas, Ponce, PRI; 3 Allergy and Immunology, Hospital Episcopal San Lucas, Ponce, PRI

**Keywords:** autoimmune hemolytic anemia, direct antiglobulin test (dat), intravenous immunoglobulin, isohemagglutinins, ivig-associated hemolysis, kawasaki disease, non-o blood group risk

## Abstract

Autoimmune hemolytic anemia (AIHA) is an uncommon but recognized complication of intravenous immunoglobulin (IVIG) therapy in Kawasaki disease (KD). The mechanism is thought to involve passive transfer of donor isohemagglutinins that bind to recipient red blood cell (RBC) antigens, causing immune-mediated hemolysis. We describe four pediatric cases of IVIG-induced AIHA following treatment for KD.

We retrospectively reviewed four KD patients who developed hemolytic anemia temporally associated with IVIG administration. Clinical data included age, blood type, transfusion requirement, and outcomes. All four patients were non-O blood types. Hemolysis developed within days of IVIG infusion, confirmed by a positive direct antiglobulin test. Two patients required red blood cell transfusions for symptomatic anemia. The lowest recorded hemoglobin levels ranged from 6.6 to 7.9 g/dL. None required corticosteroids or additional immunosuppression. All patients achieved full recovery with iron and folate supplementation. No coronary aneurysms or long-term complications were observed.

IVIG-induced AIHA is a rare but clinically significant complication of KD therapy, particularly in non-O blood group patients. Routine post-infusion hemoglobin monitoring and early recognition of anemia are essential. Supportive management alone is typically sufficient, and prognosis remains excellent when the condition is promptly identified and managed.

## Introduction

Autoimmune hemolytic anemia (AIHA) is a rare, acquired disorder in which autoantibodies target antigens on the surface of red blood cells (RBCs), leading to hemolysis and, despite erythropoietin-driven reticulocytosis, frequently resulting in anemia [1,2]. In children, AIHA is uncommon, with an approximate annual incidence of 0.8 per 100,000 individuals under 18 years old [1]. 

AIHA is classified as "warm" or "cold" based on the temperature at which antibodies exhibit maximal reactivity. In pediatrics, warm-reactive IgG antibodies that bind optimally at 37°C are most common [1]. Diagnosis relies primarily on the direct antiglobulin test (DAT), the gold standard for detecting antibody coating on RBCs [2]. Early recognition of pediatric AIHA is critical, as it often presents acutely and can be life-threatening, requiring prompt diagnosis, treatment initiation, and close monitoring [1].

Intravenous immunoglobulin (IVIG) has also been implicated as a rare trigger for secondary AIHA in children. IVIG is a pooled blood product and the standard treatment for Kawasaki disease (KD), an acute pediatric vasculitis in which timely treatment reduces the risk of coronary artery aneurysms [3]. High-dose IVIG (2g/Kg) with aspirin significantly lowers coronary complications in KD. While IVIG is generally safe and well-tolerated, most adverse effects are infusion-related. A less common but important complication is hemolytic anemia, reported mainly in case reports and small series, which may be under-recognized [3]. 

The leading mechanism is thought to be passive transfer of anti-erythrocyte antibodies present in the IVIG preparations, leading to antibody-mediated RBC destruction. In particular, donor anti-A and anti-B IgG antibodies can coat recipient RBCs, especially in non-type O patients who cannot readily neutralize these antibodies [4]. We encountered four unique cases of children diagnosed with Kawasaki disease who were treated with one or two doses of (2g/Kg) IVIG over an 8-12 hour infusion, who developed autoimmune hemolytic anemia.

The objective of this study is to describe a case series of pediatric patients with Kawasaki disease who developed autoimmune hemolytic anemia following intravenous immunoglobulin (IVIG) therapy, highlighting clinical presentation, management, and potential mechanisms.

## Case presentation

Case one

An 11-month-old previously healthy female with blood type A+ and no significant past medical history presented with nine days of high-grade fever, extremity edema, perioral erythema, and diffuse rash, consistent with KD. She was treated with IVIG (2g/Kg) and high-dose aspirin. A baseline DAT prior to IVIG administration was negative. Although she initially improved, her hemoglobin declined to 7.8 g/dL without bleeding. A repeat DAT became positive, confirming AIHA. Despite this, she remained clinically stable and did not require a blood transfusion. The patient was managed with supportive care, including iron and folate, and fully recovered. She was discharged with low-dose aspirin and was advised for cardiology follow-up due to a small pericardial effusion detected during her hospitalization.

Case two

A three-year-old male with blood type B+ and a past medical history significant for mild autism presented with persistent fever, rash, extremity edema, and desquamation, consistent with KD. Additionally, he was diagnosed with a concurrent influenza B infection. Management included Tamiflu and two doses of IVIG (2g/Kg). Several days later, he developed pallor and fatigue, and his hemoglobin dropped from 8.2 g/dL to 7.6 g/dL, despite stable reticulocyte counts. A positive DAT confirmed the presence of immune-mediated hemolysis. The patient required a blood transfusion due to persistent anemia and was transferred to the pediatric ICU for observation and specialist evaluation. He improved after transfusion and was discharged with close outpatient follow-up to monitor for any further complications.

Case three

A 10-month-old male with blood type A+ and no significant past medical history was admitted with nine days of fever, irritability, rash, conjunctivitis, and mucosal changes, all indicative of Kawasaki disease (KD). Laboratory tests showed leukocytosis, elevated inflammatory markers, and transaminitis. After supportive care, the patient received IVIG (2 g/kg) and high-dose aspirin. Following a reduction in fever, his hemoglobin dropped from 9.9 g/dL to 6.6 g/dL, with reticulocytosis at 5.6% and a positive DAT, consistent with immune-mediated hemolysis. The patient required one RBC transfusion, and his hemoglobin rose to 11 g/dL post-transfusion. He was discharged and continued his recovery on low-dose aspirin, with cardiology and pediatric follow-up arranged to ensure no long-term cardiac complications.

Case four

A one-year-seven-month-old male with blood type A+ and no significant past medical history presented with fever and features of KD. He received IVIG (2g/Kg) along with high-dose aspirin, and later transitioned to low-dose therapy. An echocardiogram revealed preserved biventricular function, mild aortic and tricuspid regurgitation, and no coronary dilation or aneurysm, which is typical for patients with well-managed KD. Post-IVIG, the patient developed Coombs-positive hemolytic anemia (AIHA), with hemoglobin decreasing to 7.9 g/dL, a positive DAT, and reticulocytosis. He did not require a blood transfusion and showed improvement with iron and folate supplementation. The patient was discharged on low-dose aspirin and was advised for cardiology follow-up to monitor for any cardiac issues that may arise from the underlying KD.

In the four cases reviewed, clinical characteristics, laboratory findings, treatments, and outcomes are summarized in Table 1.

**Table 1:** Clinical characteristics, laboratory findings, treatments, and outcomes of the four pediatric patients who developed IVIG-associated hemolytic anemia following treatment for Kawasaki disease IVIG - intravenous immunoglobulin

Characteristic	Patient one	Patient two	Patient three	Patient four
Age / sex	11-month-old female	3-year-old male	10-month-old male	19-month-old male
Blood type	A+	B+	A+	A+
IVIG dose	2 g/kg ×1	2 g/kg ×2	2 g/kg ×1	2 g/kg ×1
Hemoglobin nadir (g/dL)	7.8	7.6	6.6	7.9
Direct antiglobulin test (DAT)	Positive	Positive	Positive	Positive
Reticulocytosis	Stable reticulocytes counts	Stable reticulocytes counts	5.60%	Stable reticulocytes counts
Symptoms	Fatigue, pallor	Pallor, fatigue	Fatigue, pallor	Pallor
Transfusion required	No	Yes (1 unit RBC)	Yes (1 unit RBC)	No
Other interventions	Iron and folate	Iron/folate; ICU observation	Iron and folate	Iron and folate
Cardiac findings	Small pericardial effusion	None	None	Mild AI/TR; no coronary dilation
Outcome	Full recovery	Full recovery	Full recovery	Full recovery

## Discussion

High-dose IVIG is highly effective in KD, reducing cardiovascular complications [5]. Hemolytic anemia after IVIG is considered uncommon. Approximately 2.5% of KD patients receiving IVIG may develop IVIG-associated hemolytic anemia. Although this adverse reaction is rare, clinician vigilance remains essential [4]. 

The pathogenesis likely involves passive transfer of donor isohemagglutinins (anti-A/anti-B IgG) that bind recipient RBC antigens, leading to immune-mediated destruction. This mechanism aligns with the higher risk observed in non-O blood groups and with higher IVIG doses [4,6]. In our series, affected children were non-O, supporting this mechanism. Moreover, KD itself may also be a predisposing factor for hemolysis, given that there is immune dysregulation in this disease [3]. Once recognized, IVIG hemolysis usually resolves with supportive care within weeks. 

Given its rarity, clinicians may not immediately recognize IVIG-induced AIHA. We recommend vigilance, especially in high-risk patients (non-O blood group and/or high IVIG doses). It is recommended to perform hemolytic screening five to seven days after infusion in patients receiving higher doses of IVIG and patients with non-O blood group [6]. Any unexpected anemia warrants a hemolysis workup with DAT to confirm an immune-mediated cause. In our cases, supportive care and iron/folate were sufficient except for two of the four patients who required blood transfusion. All patients ultimately recover without additional immunosuppression. If transfusion is needed, group O RBCs are advised to avoid further hemolysis. Fortunately, prior reports like ours show full recovery in children without sequelae. 

Our four cases are consistent with prior Kawasaki series yet also highlight some distinctions (Table 1). Recently, Cheon et al. reported seven cases of IVG-associated hemolysis in KD after a second IVIG dose [4]. In contrast to those series, where hemolysis often followed a second infusion of IVIG, in our cases, only two doses were given to one of the four patients that develop IVIG-induced hemolytic anemia. Nevertheless, the clinical picture was similar: hemoglobin dropped significantly (as low as 6-7 g/dL) and two of our patients eventually required transfusion, paralleling the severe end of prior reports [3]. Notably, all of our patients stabilized with supportive care (iron/folate and transfusion if needed), echoing recommendations that hemolysis is usually self-limited once IVIG clearance begins. 

In summary, while IVIG-induced hemolytic anemia is a very rare complication of Kawasaki disease treatment, the presented cases and literature reviews highlight consistent risk factors and generally favorable outcomes with recognition and supportive care. We encourage clinicians to monitor hemoglobin levels in KD patients receiving IVIG, particularly those with non-O blood groups, so that this potentially life-threatening hemolysis is not overlooked.

Limitations and future directions

This study has several limitations. The small sample size reflects the rarity of IVIG-induced autoimmune hemolytic anemia in Kawasaki disease, which inherently limits generalizability. As a case series, causal relationships cannot be definitively established. Additionally, variability in laboratory evaluation and limited long-term follow-up may restrict assessment of delayed hemolysis. Additionally, pre-IVIG antibody screen results were not consistently documented in our cases, and formal antibody screen panels with phase reactivity, autocontrol, and eluate testing were not performed. These represent significant limitations in the completeness of the serologic workup. Future studies and reported cases would benefit from a standardized transfusion medicine workup, including antibody screening, eluate testing, phase-reactivity analysis, and early consultation with transfusion medicine specialists. Such evaluation may help further characterize the immunohematologic mechanisms of IVIG-associated hemolysis and improve diagnostic accuracy and management strategies.

## Conclusions

IVIG-induced autoimmune hemolytic anemia is a rare but important complication in Kawasaki disease, particularly among non-O blood group patients. Our cases highlight the need for early recognition through post-infusion hemoglobin monitoring and prompt diagnostic evaluation. Despite its potential severity, the condition is typically self-limited and responds well to supportive care, with excellent outcomes. Increased clinical awareness is essential to ensure timely management and prevent unnecessary morbidity.
